# Compensatory Evolution of Net-Charge in Influenza A Virus Hemagglutinin

**DOI:** 10.1371/journal.pone.0040422

**Published:** 2012-07-12

**Authors:** Yuki Kobayashi, Yoshiyuki Suzuki

**Affiliations:** 1 Graduate School of Natural Sciences, Nagoya City University, Nagoya City, Aichi, Japan; 2 Department of Zoology, University of Oxford, Oxford, United Kingdom; Faculty of Biochemistry Biophysics and Biotechnology, Jagiellonian University, Poland

## Abstract

The propagation of influenza A virus depends on the balance between the activities of hemagglutinin (HA) for binding to host cells and neuraminidase (NA) for releasing from infected cells (HA-NA balance). Since the host cell membrane and the sialic acid receptor are negatively charged, the amino acid substitutions increasing (charge+) and decreasing (charge−) the positive charge of HA subunit 1 (HA1) enhance and reduce, respectively, the binding avidity and affinity. The positive charge of HA1 in human influenza A virus bearing subtype H3N2 (A/H3N2 virus) was observed to have increased during evolution, but the evolutionary mechanism for this observation was unclear because this may disrupt the HA-NA balance. Here we show, from the phylogenetic analysis of HA for human A/H3N2 and A/H1N1 viruses, that the relative frequencies of charge+ and charge− substitutions were elevated on the branches where the number of N-glycosylation sites (NGS) increased and decreased, respectively, compared to those where the number of NGS did not change. On the latter branches, the net-charge of HA1 appeared to have been largely maintained to preserve its structure and function. Since the charge+ and charge− substitutions in HA1 have opposite effects to the gain and loss of NGS on the binding and release of the virus, the net-charge of HA1 may have evolved to compensate for the effect of the gain and loss of NGS, probably through changing the avidity. Apparently, the relative frequency of charge− substitutions in HA1 of A/H3N2 virus was elevated after the introduction of oseltamivir, and that of charge+ substitutions in HA1 of A/H1N1 virus was elevated after the spread of oseltamivir resistance. These observations may also be explained by the compensatory effect of the net-charge in HA1 on the NA activity for keeping the HA-NA balance.

## Introduction

Influenza A virus is a segmented, negative-stranded RNA virus belonging to the family *Orthomyxoviridae.* This virus is classified into subtypes H1-16 and N1-9 based on the antigenic and genetic differences in hemagglutinin (HA) and neuraminidase (NA), respectively, which form spikes on the virion [1]. Influenza A viruses bearing subtypes H3N2 (A/H3N2 virus) and H1N1 (A/H1N1 virus) are currently co-circulating in humans causing seasonal influenza; the A/H3N2 virus emerged in the human population in 1968, whereas the A/H1N1 virus first emerged in 1918, disappeared in 1956, and re-emerged in 1977 [2].

The HA is the major envelope glycoprotein of influenza A virus. The HA consists of the signal peptide, HA1, and HA2, and forms trimers in the virion [3]. The HA binds to the sialic acid receptor of the host cell through the receptor binding pocket (RBP), which is located in the distal part of HA1 [4].

The HA1 is positively charged in general [5]. The amino acid substitutions may be divided into the “charge+”, “charge−”, and “charge±” substitutions, according to whether they increase, decrease, or maintain the positive charge, respectively. The charge+ and charge− substitutions around RBP are known to strengthen and weaken the affinity to the receptor, respectively, which is negatively charged [6]–[8]. In addition, the charge+ and charge− substitutions in HA1 enhance and reduce the avidity to the membrane, respectively, which is also negatively charged [9].

It should be noted, however, that the propagation of influenza A virus depends on the balance between the HA activity of receptor binding upon infection into host cells and the NA activity of cleaving the binding of HA and receptor upon release from infected cells (HA-NA balance) [6]. Therefore, changing the charge of HA may be deleterious to the virus unless the NA activity is modified appropriately [10]. The HA is also the major determinant of antigenicity. The antigenic sites (AS), which are the target of neutralizing antibodies (Ab), have been identified in HA1 [11].

Influenza A viruses are known to escape from the host immune responses through point mutations in the AS (antigenic drift) and reassortments of genomic segments encoding antigenic proteins (antigenic shift) [12]. In addition, the gain of N-glycosylation sites (NGS) in HA1 has been indicated to be involved in the escape because N-glycans can physically interfere with the binding of Ab to AS [13], [14]. The number of NGS in HA1 was observed to have increased continuously after the emergence of A/H3N2 virus in the human population, which was considered to represent an adaptation process of the virus to relatively strong immune responses in humans [15]–[17]. However, the gain of NGS may also exert deleterious effect because N-glycans may mask the RBP [18]–[20] and may be negatively charged due to the addition of sialic acid or sulfuric acid [21], [22].

Recently, it was proposed that amino acid changes raising the avidity are also involved in the escape, because the virus with a high avidity may bind to the host cell receptor before recognition by Ab [9]. Similarly to the case for the number of NGS, the positive charge of HA1 was observed to have increased continuously after the emergence of A/H3N2 virus in the human population, which was again considered to represent an adaptation process [5]. However, this hypothesis was questionable because increasing the positive charge itself may be deleterious to the virus, as mentioned above.

Here, it is interesting to note that the change in the positive charge of HA1 appears to have paralleled with that of the number of NGS during evolution of A/H3N2 virus [5], [15]. Since the increase in the positive charge of HA1 appears to exert an opposite effect to the gain of NGS on virus infection and release [23], [24], it is possible that the positive charge of HA1 has increased to compensate for the deleterious effect of the gain of NGS during evolution of human A/H3N2 virus.

In the phylogenetic analysis of proteins, compensatory amino acid changes are expected to be observed on the same branches as co-variations, because the occurrence of either one of these changes is deleterious and such a change is likely to be quickly eliminated from the population [25]. The purpose of the present study was to examine the evolutionary mechanisms of the net-charge in HA1 of human A/H3N2 and A/H1N1 viruses by focusing on its relationships with the number of NGS and the oseltamivir.

## Results

### Evolution of the Net-charge and the Number of NGS in HA1

**Figure 1:**
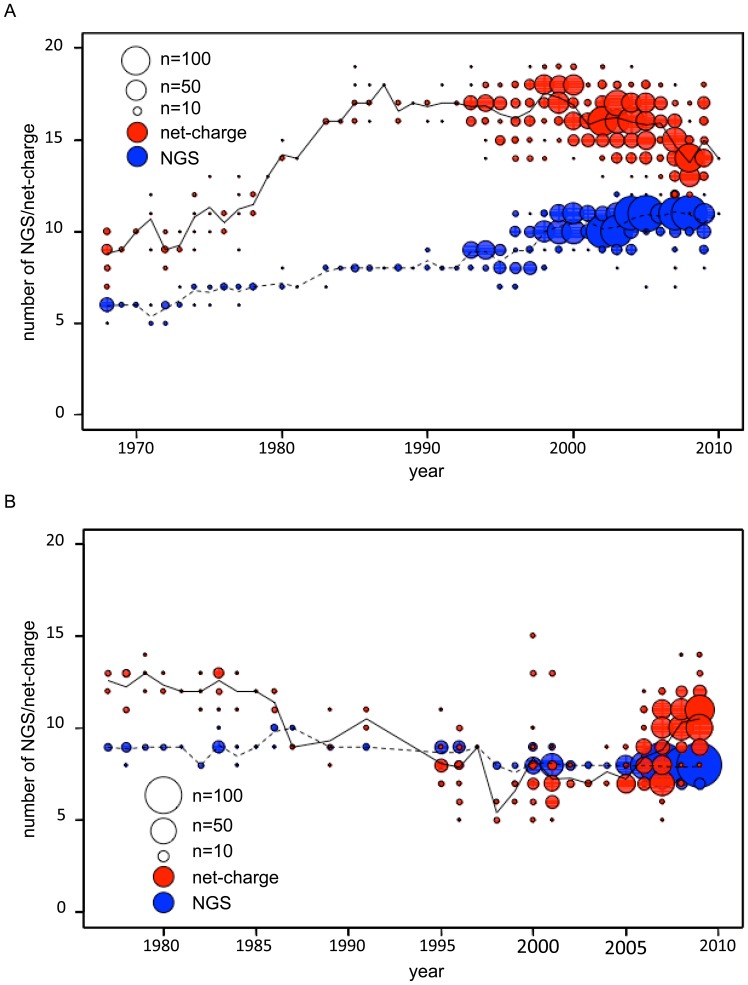
Temporal changes in the net-charge and the number of NGS in HA1. The net-charge (red circles) and the number of NGS (blue circles) in HA1 were plotted against the year of isolation for each strain of (A) A/H3N2 (*n* = 1,903) and (B) A/H1N1 (*n* = 723) viruses. The area of the circles is proportional to the frequencies of the strains, with the scales indicated at the corner. The solid and dashed lines represent the average values of the net-charge and the number of NGS in HA1 for the viruses isolated in each year, respectively.

When the net-charge and the number of NGS in HA1 were plotted against the year of isolation for each strain of A/H3N2 virus, it was observed that both of these values have increased continuously after the emergence of the virus in the human population in 1968 (Figure 1A), which was consistent with the results obtained in the previous studies [5], [15]. The number of NGS continued to increase until recently. However, the net-charge tended to decrease around the year of 2000 and later. In contrast, both the net-charge and the number of NGS were observed to have decreased continuously after the re-emergence of A/H1N1 virus in 1977 (Figure 1B). Although the number of NGS continued to decrease until recently, the net-charge tended to increase around the year of 2005 and later.

### Co-variation of the Net-charge and the Number of NGS in HA1

In the phylogenetic trees constructed for the HA of A/H3N2 and A/H1N1 viruses (Figures S1 and S2), the branches were classified into the “trunk”, “(non-trunk) interior”, and “(non-trunk) exterior” branches, where in general the effect of natural selection was considered to be reflected more weakly in this order [26], [27]. In addition, each of the trunk, interior, and exterior branches was further categorized as the “NGS+”, “NGS−”, or “NGS±” branch, where the number of NGS in HA1 was inferred to have been increased, decreased, or maintained, respectively.

**Figure 2:**
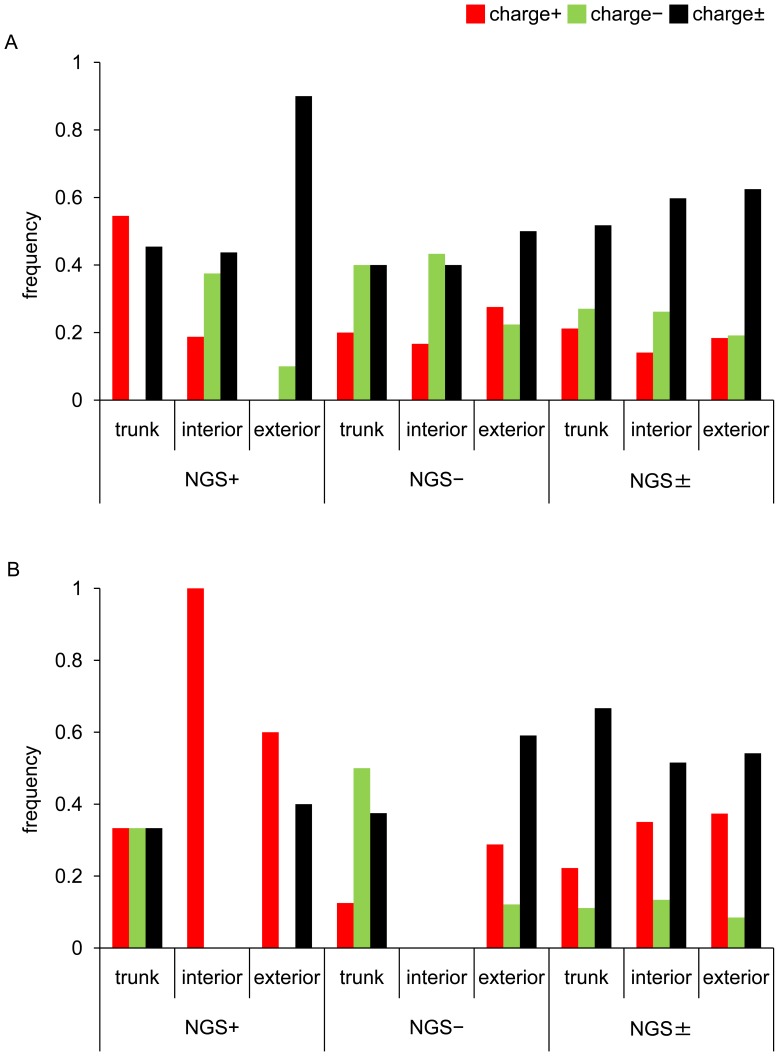
Patterns of amino acid substitutions in HA1 on different branches of phylogenetic trees. The proportions of charge+ (red bar), charge− (green bar), and charge± (black bar) substitutions in HA1 on the trunk, interior, and exterior branches of the NGS+, NGS−, and NGS± branches are indicated for (A) A/H3N2 and (B) A/H1N1 viruses. The number of amino acid substitutions in HA1 is shown in Table S1.

In the A/H3N2 virus, it was observed that the relative frequencies of charge+ and charge− substitutions were apparently elevated on the NGS+ and NGS− branches compared to the NGS± branches, respectively (Figure 2A, Table S1). When the relative frequency of charge+ to charge± substitutions was compared between the NGS+ and NGS± branches combining the data on the trunk, interior, and exterior branches, the difference was not statistically significant. However, the difference became significant by eliminating the data on the exterior branches, which were known to contain deleterious mutations (*p* = 0.0311; Fisher’s exact test). The relative frequency of charge− to charge± substitutions on the NGS− branches was significantly greater than that on the NGS± branches, irrespective of whether the data on the trunk, interior, and exterior branches were combined (*p* = 0.0177; Fisher’s exact test) or the data on the exterior branches were eliminated (*p* = 0.0374; Fisher’s exact test).

In the A/H1N1 virus, the relative frequency of charge+ to charge± substitutions on the NGS+ branches was not significantly greater than that on the NGS± branches, although there was such a tendency (Figure 2B, Table S1). However, the relative frequency of charge− to charge± substitutions on the NGS− branches was significantly greater than that on the NGS± branches when the data on the trunk and interior branches were combined (*p* = 0.0405; Fisher’s exact test).

**Figure 3:**
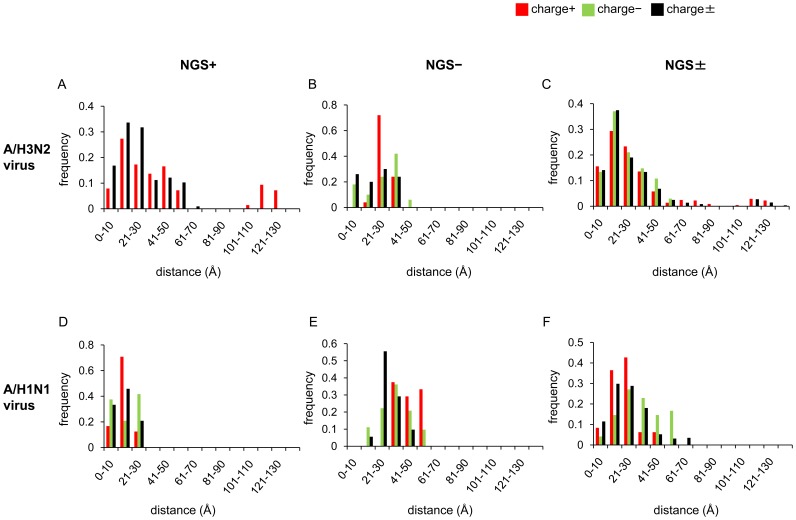
Distribution of distances from RBP to amino acid substitutions. The distances in the three-dimensional structure of HA were measured between RBP and amino acid substitutions occurring on the NGS+ (A and D), NGS− (B and E), and NGS± (C and F) trunk branches for A/H3N2 (A-C) and A/H1N1 (D-F) viruses.

To examine whether the compensatory effect of the net-charge on the gain and loss of NGS, if any, was exerted through changing the affinity to the sialic acid receptor or the avidity to the host cell membrane, the distances of charge+ and charge− substitutions in HA1 from the RBP were compared among the NGS+, NGS−, and NGS± branches in the three-dimensional structure of HA in the A/H3N2 and A/H1N1 viruses. In both viruses, there was apparently no tendency that the charge+ and charge− substitutions were more closely or distantly located from the RBP on the NGS+ and NGS− branches compared to the NGS± branches (Figures 3 and S3).

### Evolution of the Net-charge in HA1 on the NGS± Branches

**Figure 4:**
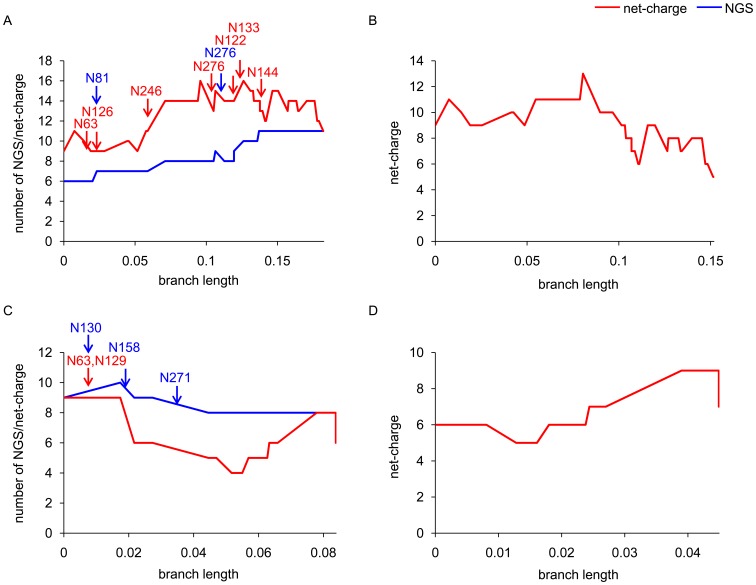
Net-charge and number of NGS in HA1 on the trunk branches. The sums of changes in the net-charge (red line) and the number of NGS (blue line) in HA1 from the root to each node along all trunk branches (A: A/H3N2 virus; C: A/H1N1 virus) and that in the net-charge along NGS± trunk branches (B: A/H3N2 virus; D: A/H1N1 virus) were plotted against the total branch length from the root to the node. The red and blue arrows indicate the trunk branches where gains and losses of NGS occurred in HA1, respectively, with the amino acid positions of NGS.

In the phylogenetic tree of HA for influenza A viruses, the effect of natural selection is known to be reflected most strongly on the trunk branches [26], [27], as mentioned above. Therefore, the compensatory effect of the net-charge on the number of NGS in HA1, if any, may be manifested as co-variation most strongly on the trunk branches. Indeed, it was evident that the overall patterns of changes in the net-charge and the number of NGS observed using all isolates of A/H3N2 and A/H1N1 viruses (Figures 1A and 1B) were recapitulated using only the trunk branches (Figures 4A and 4C). However, when only the NGS± branches were extracted from the trunk branches in the A/H3N2 virus, the increase in the positive charge observed along the original trunk branches disappeared, although the decrease observed near to the end remained (Figure 4B). Similarly, in the A/H1N1 virus, the decrease in the positive charge observed along the original trunk branches disappeared, although the increase observed near to the end remained (Figure 4D).

**Figure 5:**
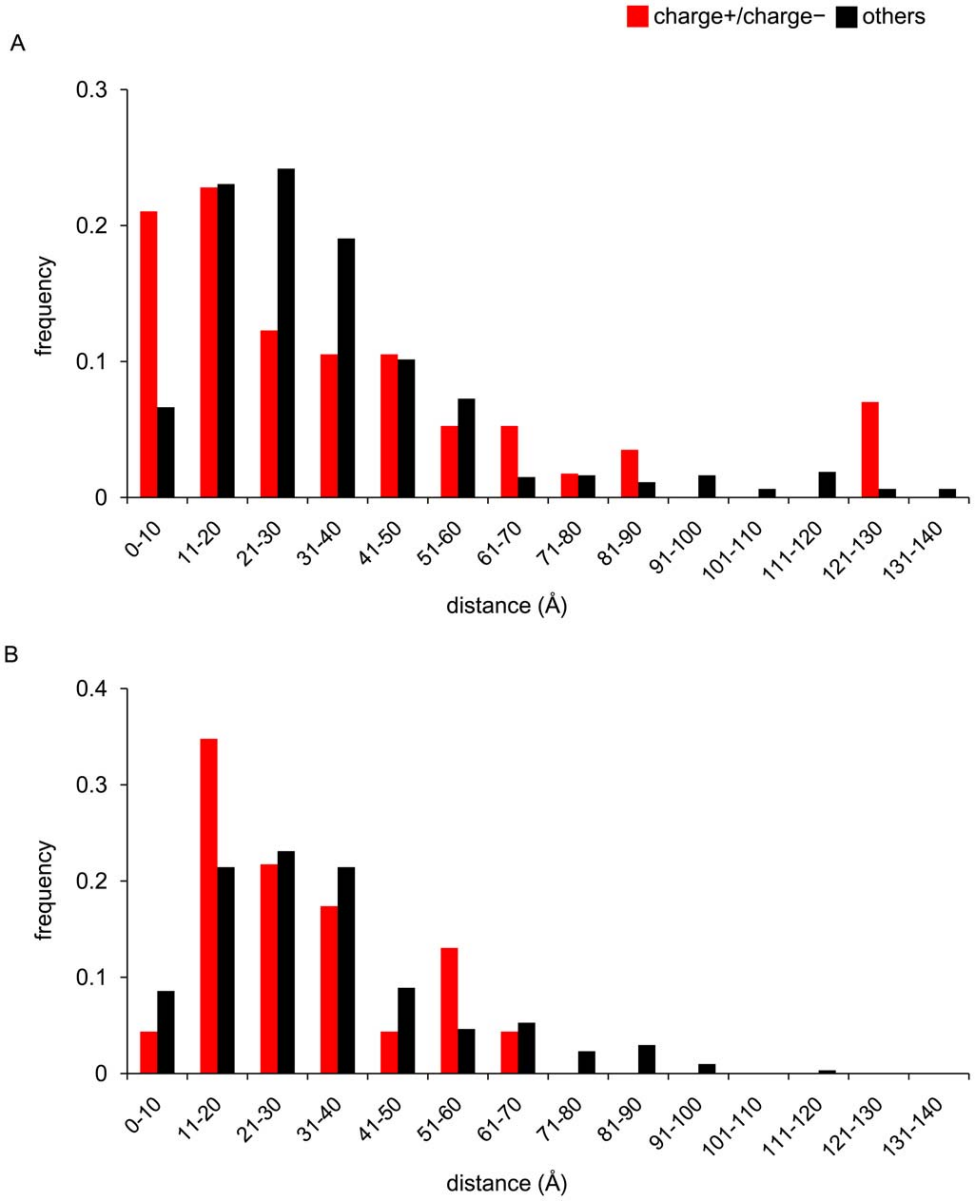
Distribution of distances between amino acid substitutions in HA1 occurring on the same NGS± branches. The distances in the three-dimensional structure of HA between amino acid substitutions occurring on the same NGS± branches were measured for (A) A/H3N2 and (B) A/H1N1 viruses. The red and black bars indicate the proportions of distances between pairs of charge+ and charge− substitutions and other pairs, respectively.

In general, proteins are known to evolve maintaining the net-charge under the structural and functional constraints, and charge+ and charge− substitutions tend to be observed as compensatory co-variations on the same branches in the phylogenetic tree and in close proximity in the three-dimensional structure of proteins [25]. Indeed, in HA1 of both the A/H3N2 and A/H1N1 viruses, the charge+ and charge− substitutions tended to be observed in more close proximity than others on the NGS± branches (Figures 5A and 5B).

### Relationship between the Net-charge in HA1 and the Oseltamivir

In HA1 of A/H3N2 virus, the positive charge appeared to have decreased around the year of 2000 and later, even though the number of NGS continued to increase (Figure 1A). In contrast, in HA1 of A/H1N1 virus, the positive charge appeared to have increased around the year of 2005 and later, even though the number of NGS continued to decrease (Figure 1B). Interestingly, the year of 2000 is close to 1999, when oseltamivir, an NA inhibitor, was introduced in humans as an anti-viral drug, and the year of 2005 is close to 2007, when oseltamivir resistance was widespread among the A/H1N1 virus [28], [29]. Oseltamivir resistance has not yet been prevalent in the A/H3N2 virus [29].

**Figure 6:**
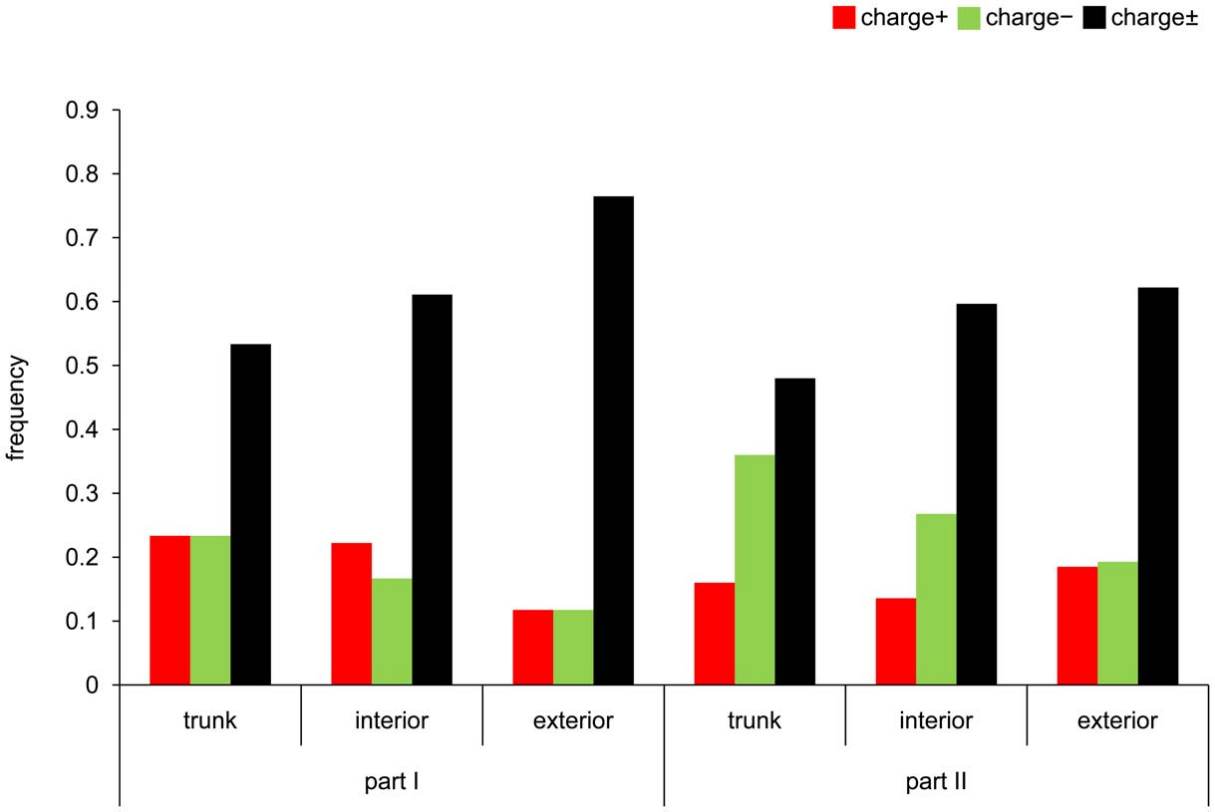
Substitution patterns before and after the introduction of oseltamivir in HA1 of A/H3N2 virus. After dividing the phylogenetic tree of A/H3N2 virus into part I and II, which roughly corresponded to the period before and after the introduction of oseltamivir in humans, respectively, the proportions of charge+ (red bar), charge− (green bar), and charge± (black bar) substitutions in HA1 on the NGS± branches were obtained for part I and II separately. The numbers of amino acid substitutions on the NGS± branches in part I and II are shown in Table S2.

To investigate the relationship between the change in the net-charge of HA1 and the introduction of oseltamivir in A/H3N2 virus, the phylogenetic tree for HA of A/H3N2 virus was divided into two parts by distinguishing the cluster containing the strains isolated after the introduction of oseltamivir (part II) from others (part I) (Figure S1). Part I contained the strains isolated in 1968-1998 and part II in 1997-2010. Part I and II roughly corresponded to the periods before and after the introduction of oseltamivir, respectively. For each part, the numbers of charge+, charge−, and charge± substitutions were counted on the NGS± branches. In part I, the numbers of charge+ and charge− substitutions were similar to each other (Figure 6, Table S2). However, in part II, the number of charge− substitutions was significantly greater than that of charge+ substitutions, irrespective of whether the data on the trunk, interior, and exterior branches were combined (*p* = 0.0231; chi-square test) or the data on the exterior branches were eliminated (*p* = 0.000183; chi-square test).

**Figure 7:**
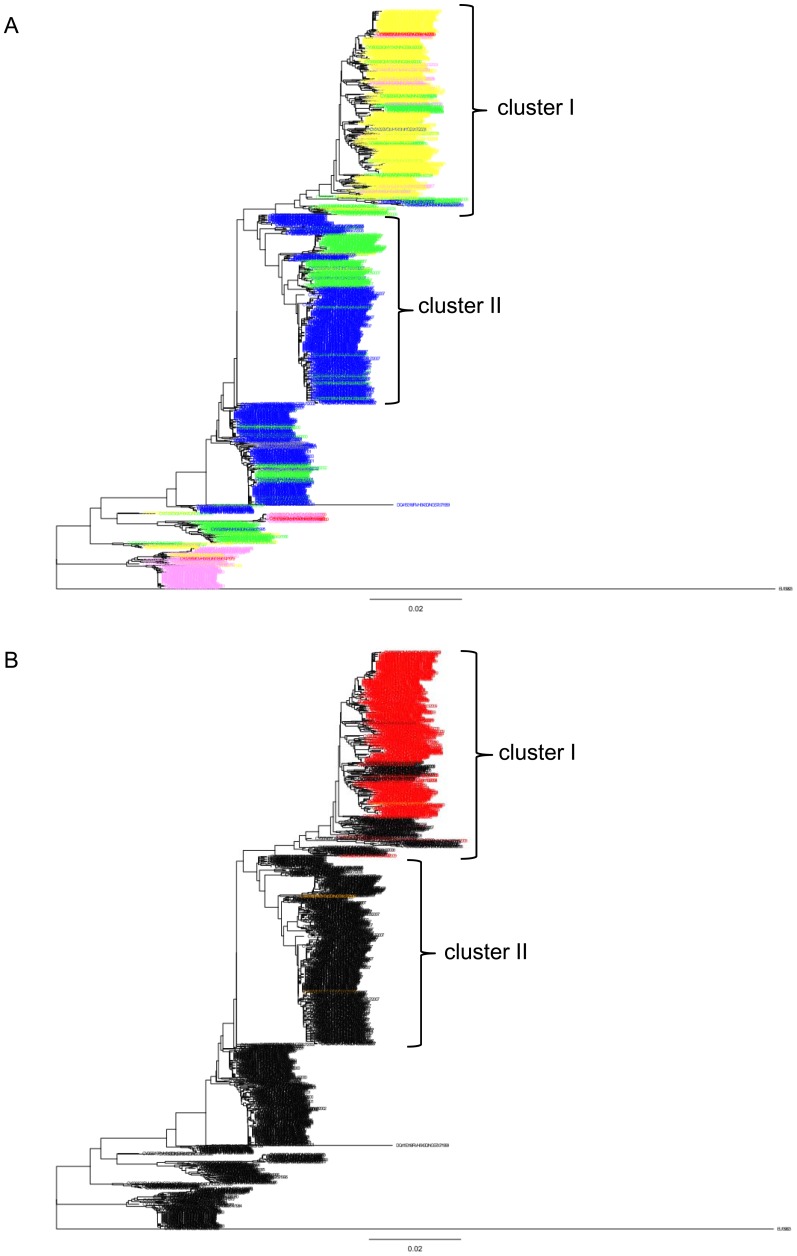
Phylogenetic tree of HA for HA-NA available strains of A/H1N1 virus. The phylogenetic tree was constructed using 998 complete HA-coding nucleotide sequences for HA-NA available strains of A/H1N1 virus. Cluster I and II correspond to those in Figure S2. (A) The strain names at exterior nodes were colored according to the net-charge in HA1; red: +14-+16, pink: +12-+13, orange: +10-+11, yellow: +8-+9, right blue: +6-+7, and blue: +4-+5. (B) The strain names at exterior nodes were colored red or orange when they had triple (Gln222, Met234, and Tyr274) or single (Tyr274) amino acid mutation in NA, respectively.

To examine the relationship between the change in the net-charge of HA1 and the widespread of oseltamivir resistance in NA of A/H1N1 virus, the HA sequences of A/H1N1 viruses whose NA sequences were also available were used to plot the net-charge in HA1 against the year of isolation. It was evident that most of the strains with elevated net-charge in HA1 after 2007 were resistant to oseltamivir (Figure S4). In the phylogenetic tree for these HA sequences, the strains isolated in 2007 or later were included in cluster I and II, which contained the strains isolated in 2002-2009 and 2002-2007, respectively (Figures 7 and S2). The net-charge of HA1 in the strains belonging to cluster I (median, +10; range, +7–+14) was greater than those belonging to cluster II (median, +7; range, +6–+10) (Figure 7A). The oseltamivir resistant strains were mainly located inside the oseltamivir sensitive strains with elevated net-charge in cluster I, which expanded after 2007 (Figure 7B).

## Discussion

In the present study, it was indicated that the net-charge has co-varied with the number of NGS in HA1 of A/H3N2 and A/H1N1 viruses after the emergence and re-emergence of these viruses in the human population until around the years of 2000 and 2005, respectively. During these periods, the charge+ and charge− substitutions appeared to have occurred more frequently on the NGS+ and NGS− branches, respectively. Since the charge+ and charge− substitutions exert opposite effects to the gain and loss of NGS on virus infection and release [6], [24], and the net-charge can be changed more easily than the number of NGS, it is likely that the charge+ and charge− substitutions have occurred to compensate for the deleterious effect of the gain and loss of NGS. It has been reported that the virus that gained NGS in HA could escape from neutralization by Ab only after the occurrence of compensatory amino acid substitutions raising the avidity [24]. Although the loss of NGS in HA1 enhances the virus infectivity, it is also problematic because it impairs the release of progeny viruses from infected cells, inducing self-aggregation at the cell surface [23], [30], [31]. This problem was solved by the occurrence of charge− substitutions in the vicinity of RBP, promoting the release of progeny viruses [23], [30], [31]. These observations suggest that keeping the balance between the binding and release (HA-NA balance) is important for productive virus replication, and the net-charge of HA1 has been used for this purpose during evolution of influenza A virus. This was also supported from the observation on the NGS± branches; the net-charge of HA1 was largely maintained to preserve its structure and function. This pattern was also observed in the H3 virus circulating in avian; both the net-charge and the number of NGS in HA1 have almost unchanged during evolution [5], [15]. Although the charge+ and charge− substitutions can alter the avidity and affinity, the compensatory effect was considered to be exerted through changing the avidity.

The co-variation of the net-charge and the number of NGS in HA1 appeared to have ceased around the years of 2000 and 2005 in A/H3N2 and A/H1N1 viruses, respectively. These years roughly corresponded to the years of 1999 and 2007, when the oseltamivir was introduced in humans and the oseltamivir resistance was widespread among the A/H1N1 virus, respectively [29]. On the NGS± branches of A/H3N2 virus, the net-charge of HA1 was maintained before the introduction of oseltamivir, but the positive charge decreased after the introduction. Since the oseltamivir resistance has not yet been prevalent in the A/H3N2 virus, it was speculated that the positive charge of HA1 may have decreased to compensate for the reduced NA activity, through enhancing the release of progeny viruses from infected cells. It has been reported that a decrease in the positive charge and a gain of NGS in HA1 reduced the sensitivity to oseltamivir by enhancing the release [32], [33]. A decrease in the positive charge of HA1 after the introduction of oseltamivir in 1999 was not evident in the A/H1N1 virus, partly because the positive charge had been decreasing continuously after the re-emergence of the virus in 1977. However, in the A/H1N1 virus, the positive charge of HA1 appeared to have increased after the oseltamivir resistance was widespread in 2007. The resistant strains possessed an elevated net-charge in HA1 apparently because they have evolved from the sensitive strains with elevated net-charge. An increase in the net-charge of HA1 may have been deleterious in the sensitive strains because it enhances only the HA activity and impairs the release of progeny viruses. Indeed, before the evolution of oseltamivir resistance, the strains with low net-charge in HA1 were predominant and those with elevated net-charge were minor. However, the oseltamivir resistance may have been able to evolve only in the strains with elevated net-charge, where the recovery of NA activity through acquisition of oseltamivir resistance may be allowed for by an enhanced HA activity. The acquisition of oseltamivir resistance may have contributed to the widespread of the strains with elevated net-charge of HA1 in the A/H1N1 virus.

It should be noted that the positive charge has increased continuously with the gain of NGS in the A/H3N2 virus, whereas it has decreased continuously with the loss of NGS in the A/H1N1 virus, before the introduction of oseltamivir. This difference may have arisen from the difference in the strength of immune responses against A/H3N2 and A/H1N1 viruses in humans. In human immunodeficiency virus type 1, it has been observed that the number of NGS in the envelope protein increased to escape from neutralization by Ab under strong immune responses in the primary-phase of infection, but decreased to enhance infectivity under weak immune responses in the end-phase of infection [34], [35]. Since positive selection was identified at the AS in A/H3N2 virus but not in A/H1N1 virus, it appears that the HA of A/H3N2 virus has evolved under strong immune responses, whereas that of A/H1N1 virus under weak immune responses [27]. These observations suggest a possibility that the number of NGS increased to escape from strong immune responses in A/H3N2 virus and decreased to enhance infectivity in A/H1N1 virus. In both viruses, the net-charge of HA1 may have evolved to compensate for the deleterious effect of gains and losses of NGS on virus infection and release.

## Materials and Methods

### Sequence Data

A total of 2,158 HA-coding nucleotide sequences of A/H3N2 virus isolated from humans in 1968-2010, and a total of 1,049 HA-coding nucleotide sequences of A/H1N1 virus isolated from humans in 1918-2009 were retrieved from the Influenza Virus Resource (http://www.ncbi.nlm.nih.gov/genomes/FLU/FLU.html) on June 15, 2010 and June 25, 2011, respectively [36]. After removing the sequences isolated from the same strains as others and those containing undetermined nucleotides and minor gaps, 1,903 complete HA-coding nucleotide sequences (1,698 nt) were retained for the analysis of A/H3N2 virus. As for the A/H1N1 virus, 754 complete HA-coding nucleotide sequences (1,695-1,698 nt) were available after removing the sequences containing undetermined nucleotides and minor gaps. Although the A/H1N1 virus circulated in humans during the periods of 1918-1956 and 1977-present, the number of sequences isolated in the former period appeared to be small (*n* = 31) for the statistical analysis, and 723 sequences isolated in the latter period (1977-2009) were used for the analysis.

### Phylogenetic Analysis

Multiple alignments of 1,903 and 723 HA-coding nucleotide sequences for A/H3N2 and A/H1N1 viruses obtained above, respectively, were made using the computer program MAFFT [37]. The optimum models of nucleotide substitutions for these sequences were selected using MODELTEST [38] with PAUP. The general time reversible model with a gamma distribution for the rate heterogeneity among sites (G) and invariable sites (I) (GTR+G+I) was selected as the optimum model for both the A/H3N2 (gamma shape parameter  = 1.3549; proportion of invariable sites  = 0.2696) and A/H1N1 (gamma shape parameter  = 1.0614; proportion of invariable sites  = 0.2763) viruses.

Phylogenetic trees for these sequences were constructed by the neighbor-joining (NJ) method with the evolutionary distance measured assuming GTR+G+I using PAUP. A/duck/Hong Kong/7/1975 (INSD accession number: CY006026) and A/swine/Iowa/15/1930 (EU139823) were used as the outgroup. The branch lengths of the phylogenetic trees were re-estimated by the maximum likelihood (ML) method with GTR+G+I. For each interior node of the phylogenetic trees, the ancestral sequence was inferred by the maximum parsimony method using PAML [39]–[41].

### Net-charge and Number of NGS

The HA-coding nucleotide sequences at the interior and exterior nodes of the phylogenetic trees were translated into the amino acid sequences. The net-charge of HA1 for each amino acid sequence (positions 1-328 and 11-328 for A/H3N2 and A/H1N1 viruses according to the H3 numbering system, respectively) was computed by subtracting the number of negatively charged residues (Asp and Glu) from that of positively charged residues (Arg, His, and Lys). The number of NGS was obtained as the number of sequons, which were defined as the sequence of Asn-Xaa-Ser/Thr, where Xaa is any amino acid except for Pro. The scatter plots of the net-charge and the number of NGS in HA1 against the year of isolation for A/H3N2 and A/H1N1 viruses were made using R.

### Classification of Branches

The branches in the phylogenetic trees were classified into the “trunk”, “(non-trunk) interior”, and “(non-trunk) exterior” branches. The trunk branches were defined as a series of consecutive branches connecting the root of the phylogenetic tree and the most recent common ancestor of the most recently isolated strains. The trunk branches consisted of interior branches and did not contain exterior branches. Each of the trunk, interior, and exterior branches was further categorized as the “NGS+”, “NGS−”, or “NGS±” branch, where the number of NGS in HA1 was inferred to have been increased, decreased, or maintained, respectively, by comparing the amino acid sequences of HA1 at the ends of the branch. It should be noted that the direction of evolution was identifiable at each branch because the phylogenetic tree was rooted.

### Counting the Number of Substitutions

For each branch of the phylogenetic trees, the numbers of charge+, charge−, and charge± substitutions were counted by comparing the amino acid sequences of HA1 at the ends of the branch. It should be noted that the substitutions causing gains and losses of NGS themselves sometimes caused changes in the charge. However, these changes were eliminated from the analysis to avoid a spurious correlation between the net-charge and the number of NGS.

### Trunk Branch Analysis

The changes in the net-charge and the number of NGS in HA1 were traced on the trunk branches of the phylogenetic trees. The sums of the changes in the net-charge and the number of NGS in HA1 from the root to each node along the trunk branches were plotted against the total branch length from the root to the node. In addition, to eliminate the effect of changes in the number of NGS on the evolution of the net-charge, only the NGS± branches were extracted from the trunk branches and were concatenated. The sum of the changes in the net-charge from the root to each node along the concatenated branches was plotted against the total branch length from the root to the node.

### Distance in the Three-dimensional Structure of HA

The distance between a pair of amino acids in the three-dimensional structure of HA was measured using the structures of A/Aichi/2/1968 (PDB ID: 3HMG) and A/Puerto Rico/8/1934 (PDB ID: 1RU7) for A/H3N2 and A/H1N1 viruses, respectively. For each amino acid, the position of the center of gravity was determined by averaging the coordinates of all atoms included. The distance between a pair of amino acids was measured as the Euclidean distance between the centers of gravity.

When the distance between amino acid substitutions and RBP was examined, the distribution of distances was constructed using all possible pairs of the amino acids where substitutions occurred and the amino acids included in the RBP (Try98, Trp153, His183, Tyr195, 130-loop (positions 135-138), 220-loop (positions 221–228), and 190-α-helix (positions 190–198)). When the distance between amino acid substitutions observed on the same branches of the phylogenetic trees was examined, the distribution of distances was constructed using all possible pairs of the amino acids where substitutions occurred.

### HA-NA Available Strains of A/H1N1 Virus

A total of 1,113 sets of HA-coding and NA-coding nucleotide sequences for the same strains of A/H1N1 virus isolated from humans in 1918-2009 were retrieved from the Influenza Virus Resource on Jan 12, 2012. After removing the sets of sequences containing undetermined nucleotides and minor gaps and those isolated in 1918-1956, 998 sets of complete HA-coding and NA-coding nucleotide sequences isolated in 1977-2009 were used for the analysis.

Phylogenetic tree for 998 HA-coding nucleotide sequences obtained above was constructed by the NJ method with the maximum composite likelihood distance using MEGA [42]. A/swine/Iowa/15/1930 was used as the outgroup. In the A/H1N1 virus, the amino acid mutation H274Y in NA is known to confer oseltamivir resistance, although R222Q and V234M are also known to be required as permissive mutations to compensate for the deleterious effect of H274Y (amino acid positions are numbered according to the N2 numbering system) [28].

## Supporting Information

Figure S1 **Phylogenetic tree of HA for A/H3N2 virus.** The phylogenetic tree was constructed using 1,903 complete HA-coding nucleotide sequences of human A/H3N2 virus. The trunk branches where the positive charge of HA1 increased and decreased were colored red and green, respectively. The numbers on the trunk branches indicate the direction and magnitude of changes in the net-charge of HA1. The numbers in parentheses indicate the direction and magnitude of changes in the net-charge of HA1 obtained when the changes due to the amino acid substitutions causing gains and losses of NGS were ignored. The red and blue arrows indicate the trunk branches where gains and losses of NGS in HA1 occurred, respectively, with the amino acid positions of NGS. The phylogenetic tree was divided into two parts by distinguishing the cluster containing the strains isolated after the introduction of oseltamivir (part II) from others (part I). The branches categorized into part II are surrounded by a dotted rectangle.
(PPTX)

Figure S2 **Phylogenetic tree of HA for A/H1N1 virus.** The phylogenetic tree was constructed using 723 complete HA-coding nucleotide sequences of human A/H1N1 virus. The trunk branches where the positive charge of HA1 increased and decreased were colored red and green, respectively. The numbers on the trunk branches indicate the direction and magnitude of changes in the net-charge of HA1. The numbers in parentheses indicate the direction and magnitude of changes in the net-charge of HA1 obtained when the changes due to the amino acid substitutions causing gains and losses of NGS were ignored. The red and blue arrows indicate the trunk branches where gains and losses of NGS in HA1 occurred, respectively, with the amino acid positions of NGS. The cluster I and II contain the strains isolated in 2002-2009 and 2002-2007, respectively.
(PPTX)

Figure S3 **Distribution of distances from RBP to amino acid substitutions.** The distances in the three-dimensional structure of HA were measured between RBP and amino acid substitutions occurring on the NGS+ (A, D, G, and J), NGS− (B, E, H, and K), and NGS± (C, F, I, and L) branches of the interior (A-F) and exterior (G-L) branches for A/H3N2 (A-C and G-I) and A/H1N1 (D-F and J-L) viruses. In (E), N.A. denotes not applicable because of the occurrence of no amino acid substitution on the NGS− interior branches for A/H1N1 virus.
(PPTX)

Figure S4 **Temporal change in the net-charge of HA1 for HA-NA available strains of A/H1N1 virus.** The net-charge of HA1 was plotted against the year of isolation for each of HA-NA available strains of A/H1N1 virus (*n* = 998). The circles were colored blue when the strains were judged as oseltamivir sensitive, and red or orange when the strains were judged as oseltamivir resistant having triple (Gln222, Met234, and Tyr274) or single (Tyr274) amino acid mutation in NA, respectively. The area of the circles is proportional to the frequencies of the strains, with the scales indicated at the corner.
(PPTX)

Table S1 **Numbers of NGS+, NGS−, and NGS± substitutions in HA1 on trunk, interior, and exterior branches of the phylogenetic trees for A/H3N2 and A/H1N1 viruses.** ^a^Charge+, charge−, and charge± denote the amino acid substitutions increasing, decreasing, and maintaining the positive charge of HA1, respectively. ^b^NGS+, NGS−, and NGS± denote the branches where the number of NGS in HA1 was inferred to have been increased, decreased, and maintained, respectively. Amino acid substitutions causing gains and losses of NGS were eliminated from counting of substitutions.
(XLSX)

Table S2 **Numbers of NGS+, NGS−, and NGS± substitutions in HA1 on NGS± branches in part I and II of the phylogenetic tree for A/H3N2 virus.** ^a^Charge+, charge−, and charge± denote the amino acid substitutions increasing, decreasing, and maintaining the positive charge of HA1, respectively. ^b^The phylogenetic tree for A/H3N2 virus was divided into part I and II as shown in Figure S1. Amino acid substitutions causing gains and losses of NGS were eliminated from counting of substitutions.
(XLSX)
