# Supplementary figures and images for: Compensatory Evolution of Net-Charge in Influenza A Virus Hemagglutinin
Source: PLoS One. 2012 Jul 12;7(7):e40422. doi: 10.1371/journal.pone.0040422 (PMC3395715; doi:10.1371/journal.pone.0040422)

## Slide 1
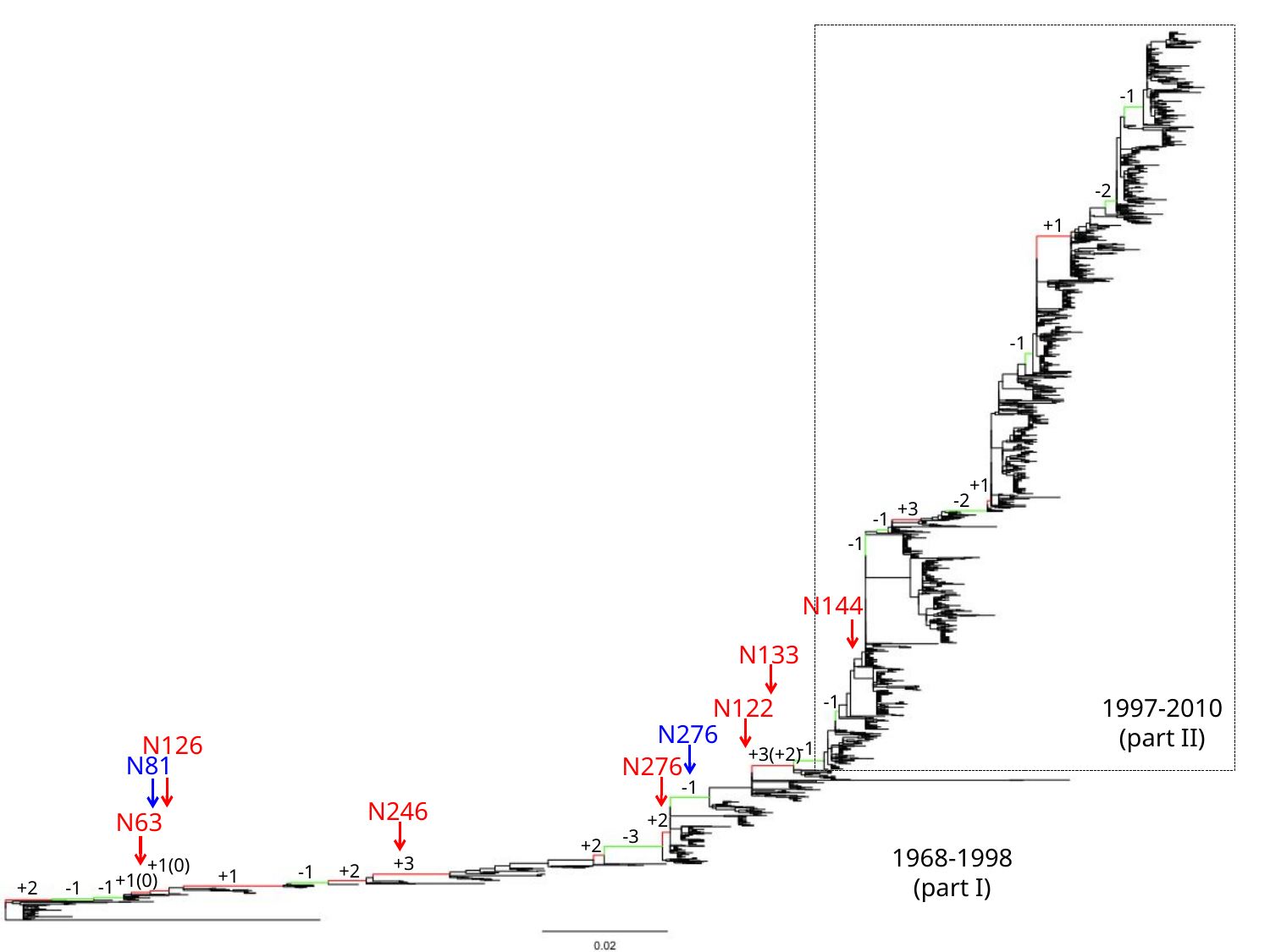

-1
-2
+1
-1
+1
-2
+3
-1
-1
N144
N133
-1
1997-2010
(part II)
N122
N276
N126
-1
+3(+2)
N81
N276
-1
N246
N63
+2
-3
+2
1968-1998
(part I)
+3
+1(0)
+2
-1
+1
+1(0)
-1
-1
+2

Supplement: Figure S1 — Phylogenetic tree of HA for A/H3N2 virus. The phylogenetic tree was constructed using 1,903 complete HA-coding nucleotide sequences of human A/H3N2 virus. The trunk branches where the positive charge of HA1 increased and decreased were colored red and green, respectively. The numbers on the trunk branches indicate the direction and magnitude of changes in the net-charge of HA1. The numbers in parentheses indicate the direction and magnitude of changes in the net-charge of HA1 obtained when the changes due to the amino acid substitutions causing gains and losses of NGS were ignored. The red and blue arrows indicate the trunk branches where gains and losses of NGS in HA1 occurred, respectively, with the amino acid positions of NGS. The phylogenetic tree was divided into two parts by distinguishing the cluster containing the strains isolated after the introduction of oseltamivir (part II) from others (part I). The branches categorized into part II are surrounded by a dotted rectangle. (PPTX) [file pone.0040422.s001.pptx]

## Slide 1
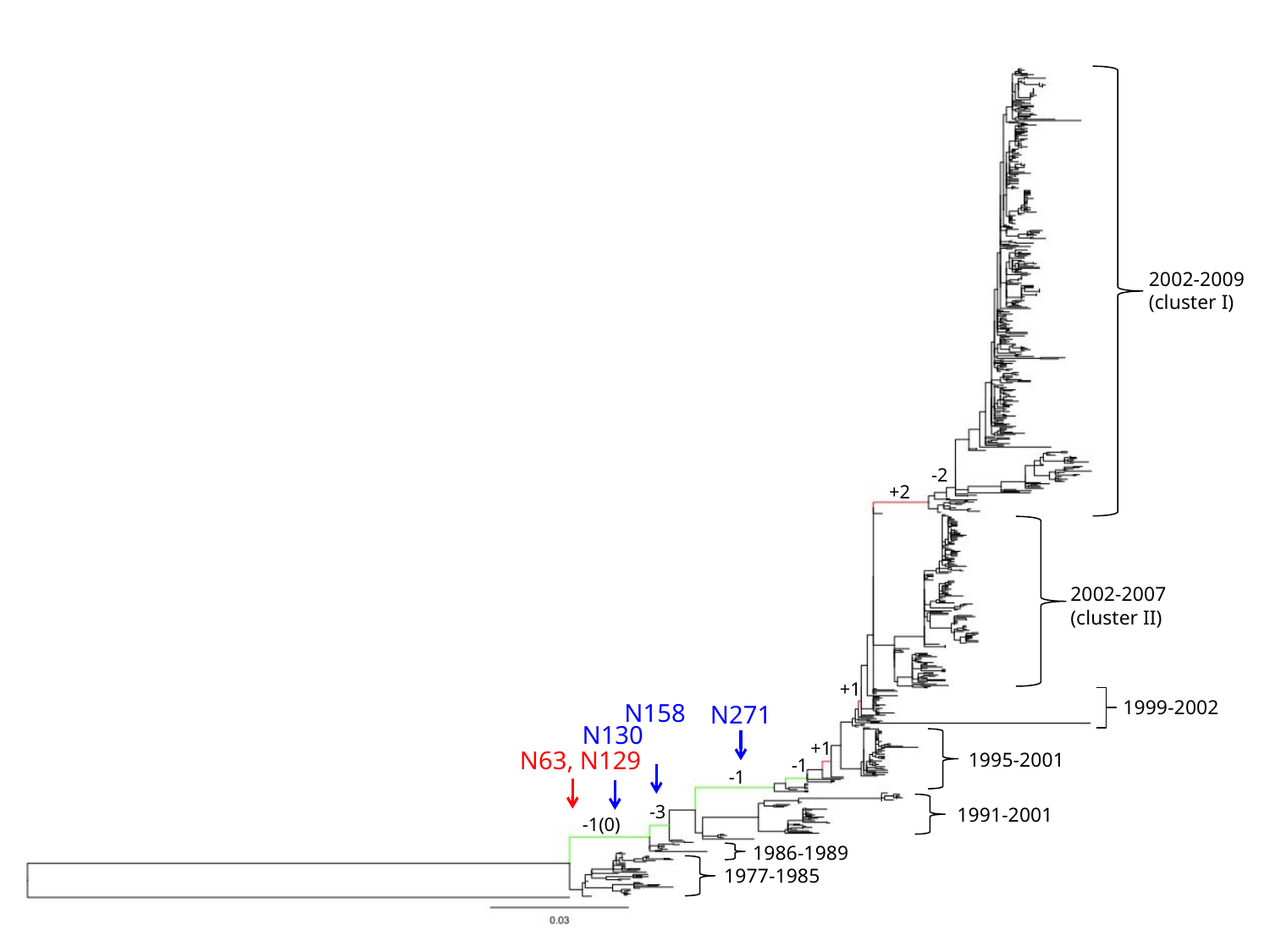

2002-2009
(cluster I)
-2
+2
2002-2007
(cluster II)
+1
1999-2002
N158
N271
N130
+1
N63, N129
1995-2001
-1
-1
-3
1991-2001
-1(0)
1986-1989
1977-1985

Supplement: Figure S2 — Phylogenetic tree of HA for A/H1N1 virus. The phylogenetic tree was constructed using 723 complete HA-coding nucleotide sequences of human A/H1N1 virus. The trunk branches where the positive charge of HA1 increased and decreased were colored red and green, respectively. The numbers on the trunk branches indicate the direction and magnitude of changes in the net-charge of HA1. The numbers in parentheses indicate the direction and magnitude of changes in the net-charge of HA1 obtained when the changes due to the amino acid substitutions causing gains and losses of NGS were ignored. The red and blue arrows indicate the trunk branches where gains and losses of NGS in HA1 occurred, respectively, with the amino acid positions of NGS. The cluster I and II contain the strains isolated in 2002-2009 and 2002-2007, respectively. (PPTX) [file pone.0040422.s002.pptx]
