# Supplementary material for: Compensatory Evolution of Net-Charge in Influenza A Virus Hemagglutinin
Source: PLoS One. 2012 Jul 12;7(7):e40422. doi: 10.1371/journal.pone.0040422 (PMC3395715; doi:10.1371/journal.pone.0040422)
Supplement: Figure S3 — Distribution of distances from RBP to amino acid substitutions. The distances in the three-dimensional structure of HA were measured between RBP and amino acid substitutions occurring on the NGS+ (A, D, G, and J), NGS− (B, E, H, and K), and NGS± (C, F, I, and L) branches of the interior (A-F) and exterior (G-L) branches for A/H3N2 (A-C and G-I) and A/H1N1 (D-F and J-L) viruses. In (E), N.A. denotes not applicable because of the occurrence of no amino acid substitution on the NGS− interior branches for A/H1N1 virus. (PPTX) [file pone.0040422.s003.pptx]

## Slide 1
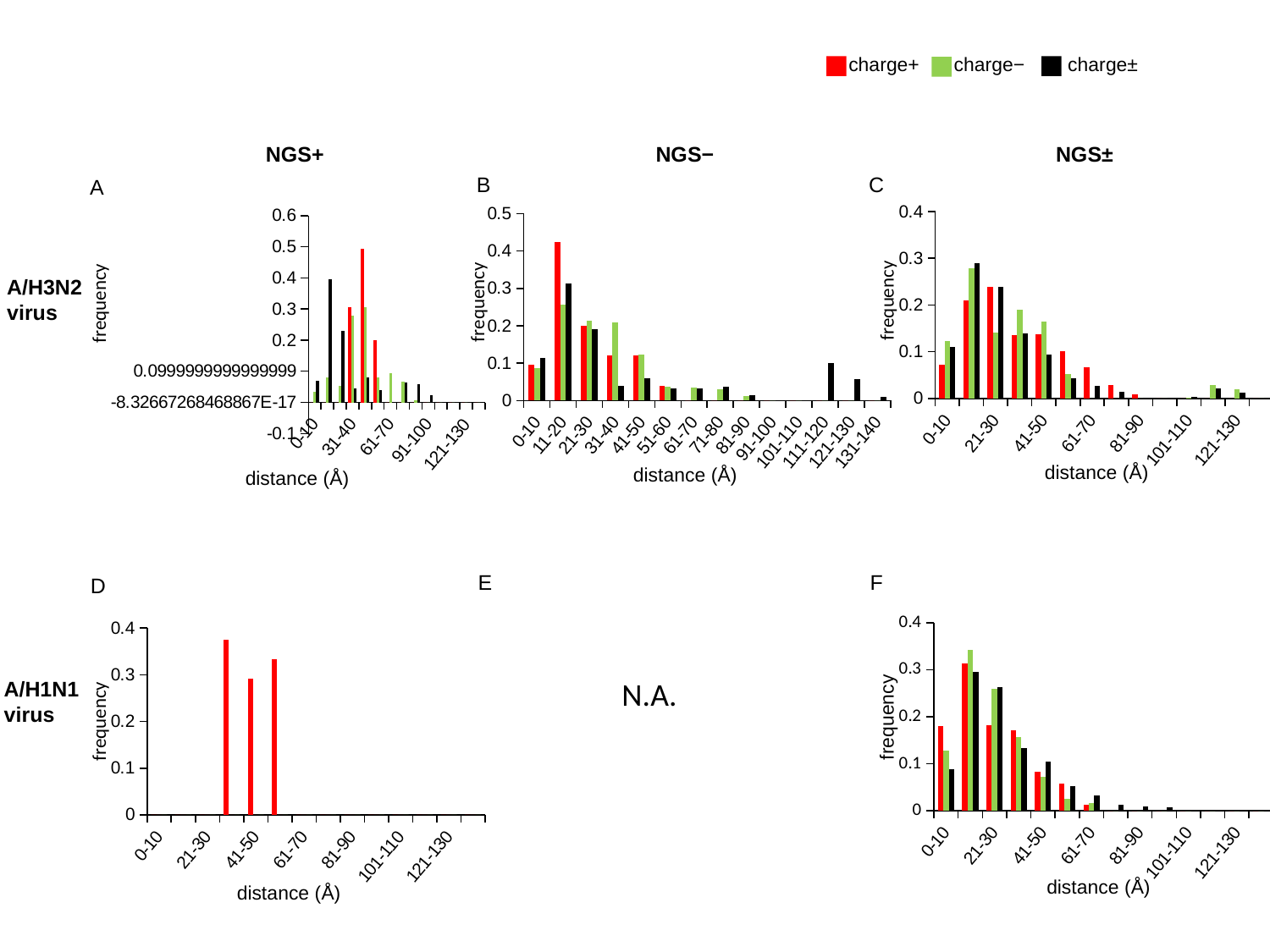

charge+
charge−
charge±
NGS+
NGS−
NGS±
B
### Chart
| Category | positive-RBS | negative-neutral | neutral-RBS |
|---|---|---|---|
| 0-10 | 0.096 | 0.0861538461538463 | 0.113333333333333 |
| 11-20 | 0.424 | 0.255384615384616 | 0.313333333333333 |
| 21-30 | 0.2 | 0.212307692307692 | 0.19 |
| 31-40 | 0.12 | 0.209230769230769 | 0.04 |
| 41-50 | 0.12 | 0.123076923076923 | 0.06 |
| 51-60 | 0.04 | 0.036923076923077 | 0.0333333333333333 |
| 61-70 | 0.0 | 0.0338461538461538 | 0.0333333333333333 |
| 71-80 | 0.0 | 0.0307692307692308 | 0.0366666666666667 |
| 81-90 | 0.0 | 0.0123076923076923 | 0.0133333333333333 |
| 91-100 | 0.0 | 0.0 | 0.0 |
| 101-110 | 0.0 | 0.0 | 0.0 |
| 111-120 | 0.0 | 0.0 | 0.1 |
| 121-130 | 0.0 | 0.0 | 0.0566666666666667 |
| 131-140 | 0.0 | 0.0 | 0.01 |frequency
distance (Å)
E
N.A.
C
### Chart
| Category | positive-RBS | negative-neutral | neutral-RBS |
|---|---|---|---|
| 0-10 | 0.0723809523809524 | 0.123076923076923 | 0.110786516853933 |
| 11-20 | 0.210476190476191 | 0.277948717948718 | 0.290337078651685 |
| 21-30 | 0.238095238095238 | 0.140512820512821 | 0.239550561797753 |
| 31-40 | 0.135238095238095 | 0.18974358974359 | 0.139550561797753 |
| 41-50 | 0.138095238095238 | 0.164615384615385 | 0.0934831460674157 |
| 51-60 | 0.101904761904762 | 0.0528205128205128 | 0.0435955056179775 |
| 61-70 | 0.0666666666666667 | 0.0 | 0.0269662921348314 |
| 71-80 | 0.0285714285714286 | 0.0 | 0.0141573033707865 |
| 81-90 | 0.00857142857142857 | 0.0 | 0.00224719101123596 |
| 91-100 | 0.0 | 0.0 | 0.0 |
| 101-110 | 0.0 | 0.00205128205128205 | 0.00292134831460674 |
| 111-120 | 0.0 | 0.0287179487179487 | 0.0220224719101124 |
| 121-130 | 0.0 | 0.0205128205128205 | 0.0123595505617978 |
| 131-140 | 0.0 | 0.0 | 0.00202247191011236 |frequency
distance (Å)
F
### Chart
| Category | positive-RBS | negative-neutral | neutral-RBS |
|---|---|---|---|
| 0-10 | 0.180147058823529 | 0.128205128205128 | 0.0891666666666668 |
| 11-20 | 0.3125 | 0.341346153846154 | 0.295833333333333 |
| 21-30 | 0.181985294117647 | 0.259615384615385 | 0.262083333333333 |
| 31-40 | 0.170955882352941 | 0.157051282051282 | 0.134166666666667 |
| 41-50 | 0.0833333333333333 | 0.0721153846153846 | 0.105416666666667 |
| 51-60 | 0.0575980392156863 | 0.0256410256410257 | 0.0516666666666667 |
| 61-70 | 0.0134803921568627 | 0.016025641025641 | 0.0325 |
| 71-80 | 0.0 | 0.0 | 0.0133333333333333 |
| 81-90 | 0.0 | 0.0 | 0.00875 |
| 91-100 | 0.0 | 0.0 | 0.00708333333333334 |
| 101-110 | 0.0 | 0.0 | 0.0 |
| 111-120 | 0.0 | 0.0 | 0.0 |
| 121-130 | 0.0 | 0.0 | 0.0 |
| 131-140 | 0.0 | 0.0 | 0.0 |frequency
distance (Å)
A
### Chart
| Category | positive-RBS | negative-neutral | neutral-RBS |
|---|---|---|---|
| 0-10 | 0.0 | 0.0333333333333333 | 0.0685714285714286 |
| 11-20 | 0.0 | 0.08 | 0.394285714285715 |
| 21-30 | 0.0 | 0.0533333333333334 | 0.228571428571429 |
| 31-40 | 0.306666666666667 | 0.28 | 0.0457142857142857 |
| 41-50 | 0.493333333333333 | 0.306666666666667 | 0.08 |
| 51-60 | 0.2 | 0.08 | 0.04 |
| 61-70 | 0.0 | 0.0933333333333334 | 0.0 |
| 71-80 | 0.0 | 0.0666666666666667 | 0.0628571428571429 |
| 81-90 | 0.0 | 0.00666666666666667 | 0.0571428571428571 |
| 91-100 | 0.0 | 0.0 | 0.0228571428571429 |
| 101-110 | 0.0 | 0.0 | 0.0 |
| 111-120 | 0.0 | 0.0 | 0.0 |
| 121-130 | 0.0 | 0.0 | 0.0 |
| 131-140 | 0.0 | 0.0 | 0.0 |frequency
distance (Å)
D
### Chart
| Category | positive-RBS | negative-neutral | neutral-RBS |
|---|---|---|---|
| 0-10 | 0.0 | 0.0 | 0.0 |
| 11-20 | 0.0 | 0.0 | 0.0 |
| 21-30 | 0.0 | 0.0 | 0.0 |
| 31-40 | 0.375 | 0.0 | 0.0 |
| 41-50 | 0.291666666666667 | 0.0 | 0.0 |
| 51-60 | 0.333333333333333 | 0.0 | 0.0 |
| 61-70 | 0.0 | 0.0 | 0.0 |
| 71-80 | 0.0 | 0.0 | 0.0 |
| 81-90 | 0.0 | 0.0 | 0.0 |
| 91-100 | 0.0 | 0.0 | 0.0 |
| 101-110 | 0.0 | 0.0 | 0.0 |
| 111-120 | 0.0 | 0.0 | 0.0 |
| 121-130 | 0.0 | 0.0 | 0.0 |
| 131-140 | 0.0 | 0.0 | 0.0 |frequency
distance (Å)
A/H3N2
virus
A/H1N1
virus

## Slide 2
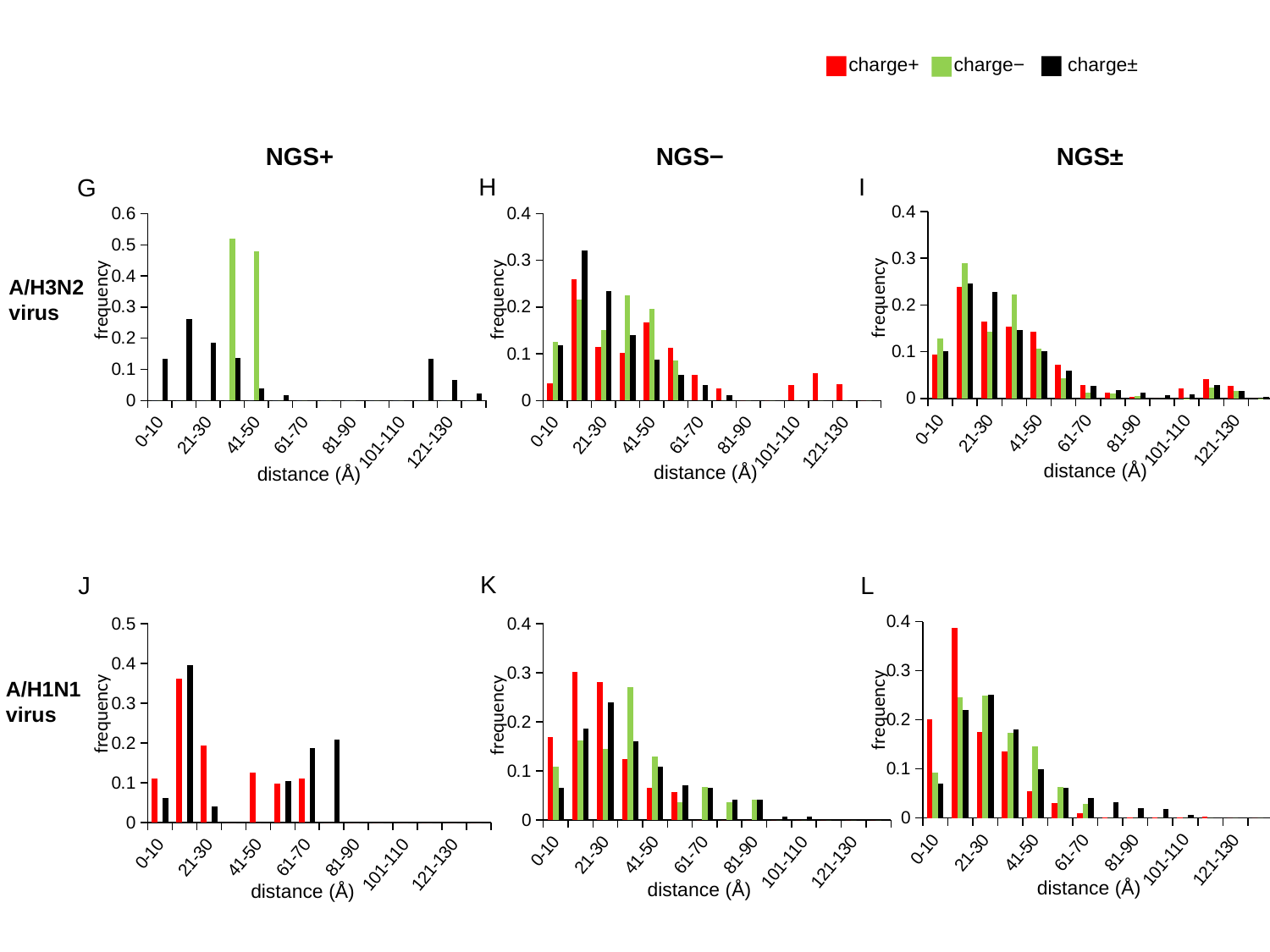

charge+
charge−
charge±
NGS+
NGS−
NGS±
H
### Chart
| Category | positive-RBS | negative-neutral | neutral-RBS |
|---|---|---|---|
| 0-10 | 0.0375 | 0.126153846153846 | 0.118709677419355 |
| 11-20 | 0.26 | 0.215384615384615 | 0.321290322580645 |
| 21-30 | 0.115 | 0.150769230769231 | 0.233548387096774 |
| 31-40 | 0.1025 | 0.224615384615385 | 0.139354838709677 |
| 41-50 | 0.1675 | 0.196923076923077 | 0.087741935483871 |
| 51-60 | 0.1125 | 0.0861538461538463 | 0.0541935483870968 |
| 61-70 | 0.055 | 0.0 | 0.0322580645161291 |
| 71-80 | 0.025 | 0.0 | 0.0116129032258065 |
| 81-90 | 0.0 | 0.0 | 0.00129032258064516 |
| 91-100 | 0.0 | 0.0 | 0.0 |
| 101-110 | 0.0325 | 0.0 | 0.0 |
| 111-120 | 0.0575 | 0.0 | 0.0 |
| 121-130 | 0.035 | 0.0 | 0.0 |
| 131-140 | 0.0 | 0.0 | 0.0 |frequency
distance (Å)
K
### Chart
| Category | positive-RBS | negative-neutral | neutral-RBS |
|---|---|---|---|
| 0-10 | 0.168859649122807 | 0.109375 | 0.0662393162393163 |
| 11-20 | 0.302631578947368 | 0.161458333333333 | 0.186965811965812 |
| 21-30 | 0.280701754385965 | 0.145833333333333 | 0.240384615384615 |
| 31-40 | 0.125 | 0.270833333333333 | 0.161324786324786 |
| 41-50 | 0.0657894736842105 | 0.130208333333333 | 0.108974358974359 |
| 51-60 | 0.0570175438596492 | 0.0364583333333333 | 0.0715811965811966 |
| 61-70 | 0.0 | 0.0677083333333334 | 0.0662393162393163 |
| 71-80 | 0.0 | 0.0364583333333333 | 0.0416666666666667 |
| 81-90 | 0.0 | 0.0416666666666667 | 0.0416666666666667 |
| 91-100 | 0.0 | 0.0 | 0.00747863247863248 |
| 101-110 | 0.0 | 0.0 | 0.00747863247863248 |
| 111-120 | 0.0 | 0.0 | 0.0 |
| 121-130 | 0.0 | 0.0 | 0.0 |
| 131-140 | 0.0 | 0.0 | 0.0 |frequency
distance (Å)
I
### Chart
| Category | positive-RBS | negative-neutral | neutral-RBS |
|---|---|---|---|
| 0-10 | 0.0942857142857142 | 0.127542857142857 | 0.100559440559441 |
| 11-20 | 0.238571428571429 | 0.290057142857143 | 0.245174825174825 |
| 21-30 | 0.163809523809524 | 0.142171428571429 | 0.227062937062937 |
| 31-40 | 0.154285714285714 | 0.222857142857143 | 0.146573426573427 |
| 41-50 | 0.142380952380952 | 0.106971428571429 | 0.100769230769231 |
| 51-60 | 0.0719047619047619 | 0.0434285714285714 | 0.0585314685314685 |
| 61-70 | 0.0292857142857143 | 0.0128 | 0.0267832167832168 |
| 71-80 | 0.0126190476190476 | 0.00982857142857142 | 0.0186713286713287 |
| 81-90 | 0.00357142857142857 | 0.00434285714285714 | 0.0116083916083916 |
| 91-100 | 0.0 | 0.0 | 0.00713286713286714 |
| 101-110 | 0.0207142857142857 | 0.000457142857142857 | 0.00902097902097902 |
| 111-120 | 0.0421428571428571 | 0.0235428571428571 | 0.0293706293706294 |
| 121-130 | 0.0264285714285714 | 0.0157714285714286 | 0.0153146853146853 |
| 131-140 | 0.0 | 0.000228571428571429 | 0.00342657342657343 |frequency
distance (Å)
L
### Chart
| Category | positive-RBS | negative-neutral | neutral-RBS |
|---|---|---|---|
| 0-10 | 0.200533661740558 | 0.0932971014492753 | 0.0701530612244898 |
| 11-20 | 0.386494252873563 | 0.246376811594203 | 0.219954648526077 |
| 21-30 | 0.175697865353038 | 0.249094202898551 | 0.251417233560091 |
| 31-40 | 0.135057471264368 | 0.173913043478261 | 0.180130385487529 |
| 41-50 | 0.054392446633826 | 0.146739130434783 | 0.0999149659863947 |
| 51-60 | 0.0303776683087028 | 0.0625 | 0.0609410430839002 |
| 61-70 | 0.0100574712643678 | 0.0280797101449276 | 0.0408163265306123 |
| 71-80 | 0.00102627257799672 | 0.0 | 0.0320294784580499 |
| 81-90 | 0.0014367816091954 | 0.0 | 0.0196995464852608 |
| 91-100 | 0.00102627257799672 | 0.0 | 0.018140589569161 |
| 101-110 | 0.00184729064039409 | 0.0 | 0.00581065759637188 |
| 111-120 | 0.00205254515599343 | 0.0 | 0.000992063492063493 |
| 121-130 | 0.0 | 0.0 | 0.0 |
| 131-140 | 0.0 | 0.0 | 0.0 |frequency
distance (Å)
G
### Chart
| Category | positive-RBS | negative-neutral | neutral-RBS |
|---|---|---|---|
| 0-10 | 0.0 | 0.0 | 0.133333333333333 |
| 11-20 | 0.0 | 0.0 | 0.262222222222222 |
| 21-30 | 0.0 | 0.0 | 0.186666666666667 |
| 31-40 | 0.0 | 0.52 | 0.137777777777778 |
| 41-50 | 0.0 | 0.48 | 0.04 |
| 51-60 | 0.0 | 0.0 | 0.0177777777777778 |
| 61-70 | 0.0 | 0.0 | 0.0 |
| 71-80 | 0.0 | 0.0 | 0.0 |
| 81-90 | 0.0 | 0.0 | 0.0 |
| 91-100 | 0.0 | 0.0 | 0.0 |
| 101-110 | 0.0 | 0.0 | 0.0 |
| 111-120 | 0.0 | 0.0 | 0.133333333333333 |
| 121-130 | 0.0 | 0.0 | 0.0666666666666667 |
| 131-140 | 0.0 | 0.0 | 0.0222222222222222 |frequency
distance (Å)
J
### Chart
| Category | positive-RBS | negative-neutral | neutral-RBS |
|---|---|---|---|
| 0-10 | 0.111111111111111 | 0.0 | 0.0625 |
| 11-20 | 0.361111111111111 | 0.0 | 0.395833333333333 |
| 21-30 | 0.194444444444445 | 0.0 | 0.0416666666666667 |
| 31-40 | 0.0 | 0.0 | 0.0 |
| 41-50 | 0.125 | 0.0 | 0.0 |
| 51-60 | 0.0972222222222222 | 0.0 | 0.104166666666667 |
| 61-70 | 0.111111111111111 | 0.0 | 0.1875 |
| 71-80 | 0.0 | 0.0 | 0.208333333333333 |
| 81-90 | 0.0 | 0.0 | 0.0 |
| 91-100 | 0.0 | 0.0 | 0.0 |
| 101-110 | 0.0 | 0.0 | 0.0 |
| 111-120 | 0.0 | 0.0 | 0.0 |
| 121-130 | 0.0 | 0.0 | 0.0 |
| 131-140 | 0.0 | 0.0 | 0.0 |frequency
distance (Å)
A/H3N2
virus
A/H1N1
virus
