# Supplementary material for: Compensatory Evolution of Net-Charge in Influenza A Virus Hemagglutinin
Source: PLoS One. 2012 Jul 12;7(7):e40422. doi: 10.1371/journal.pone.0040422 (PMC3395715; doi:10.1371/journal.pone.0040422)
Supplement: Figure S4 — Temporal change in the net-charge of HA1 for HA-NA available strains of A/H1N1 virus. The net-charge of HA1 was plotted against the year of isolation for each of HA-NA available strains of A/H1N1 virus (n = 998). The circles were colored blue when the strains were judged as oseltamivir sensitive, and red or orange when the strains were judged as oseltamivir resistant having triple (Gln222, Met234, and Tyr274) or single (Tyr274) amino acid mutation in NA, respectively. The area of the circles is proportional to the frequencies of the strains, with the scales indicated at the corner. (PPTX) [file pone.0040422.s004.pptx]

## Slide 1
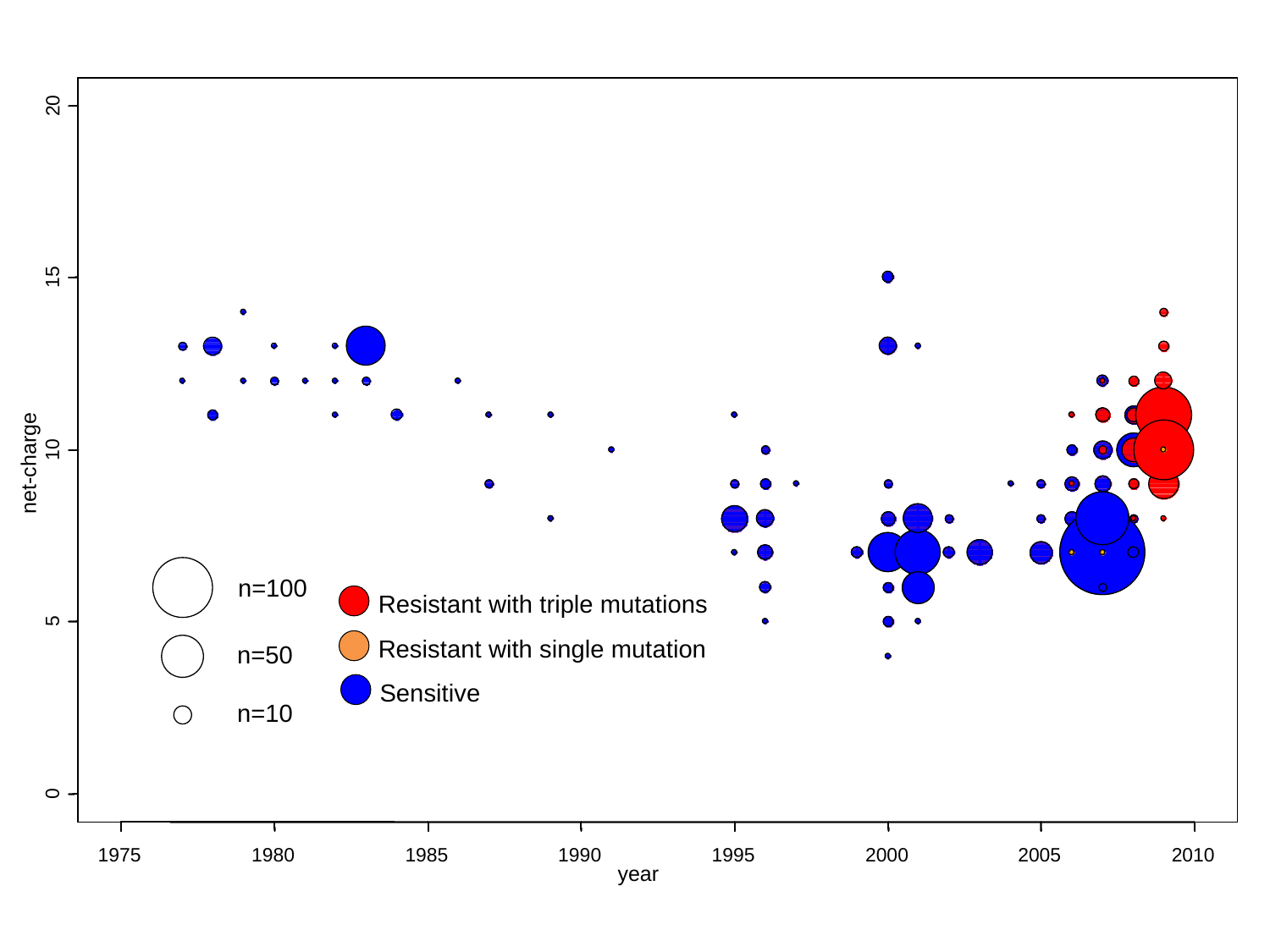

20
15
10
5
0
1975
1980
1985
1990
1995
2000
2005
2010
net-charge
n=100
Resistant with triple mutations
Resistant with single mutation
n=50
Sensitive
n=10
year
